# Environmental stress and plant-plant interactions jointly shape intertidal cordgrass traits across broad spatial scales

**DOI:** 10.3389/fpls.2026.1913369

**Published:** 2026-08-14

**Authors:** Yiwen Liu, Qian Dong, Ziyu Zheng, Wensi Hu, Yuxiang Li, Chi Xu, Shuqing N. Teng

**Affiliations:** School of Life Sciences, Nanjing University, Nanjing, China

**Keywords:** facilitation, functional traits, salt marsh, scale-dependent feedback, stress gradient hypothesis

## Abstract

In intertidal ecosystems, plant functional traits are strongly shaped by environmental stress induced by e.g. hydrodynamic disturbance and salinity. Meanwhile, local-scale plant-plant interactions (such as facilitation) can also affect plant traits to a certain extent. However, it remains unclear how these abiotic and biotic factors jointly shape plant traits across broad geographic gradients. Here, we focused on within-patch functional differentiations of a cordgrass species *Spartina alterniflora* growing patchily at low and high tidal positions located in 10 Chinese coastal sites spanning 10 latitudinal degrees. Three key functional traits of the species, namely leaf dry mass content (LDMC), specific leaf area (SLA), and plant height, were considered. Soil properties within and outside vegetation patches, as well as marine environmental variables (salinity, dissolved oxygen, nitrate and phosphate concentrations), were included as predictors in linear regression analyses. As the response variable, a trait lnRR was calculated as the logarithm ratio of the trait value at the center to that at the edge of a vegetation patch, representing within-patch facilitation strength. We found that at high tidal positions, where hydrodynamic stress was relatively weak, plants at patch centers had 15.6% higher LDMC and 13.3% lower SLA than those at patch edges. By contrast, at low tidal positions, center plants were 13.4% taller than edge plants. At both tidal positions, soil properties explained the greatest (*R*² = 0.670 and 0.479 at low and high tidal positions, respectively) proportion of variations in plant height lnRR and the least (*R*² = 0.079 and 0.105 at low and high tidal positions, respectively) variations in SLA lnRR. Across all sites, plant height lnRR increased with salinity (*r* = 0.79, *p* < 0.001) but decreased with dissolved oxygen (*r* = -0.61, *p* = 0.004) and nitrate concentration (*r* = -0.78, *p* < 0.001), reflecting greater center–edge height differences under harsher environmental conditions. These findings suggest that within-patch functional trait differences can broadly provide a signature of local facilitation, with facilitative effects becoming more pronounced under more stressful intertidal conditions. Our work not only advances our understanding of how patch-forming coastal plants organize functional variation under spatially heterogeneous and changing intertidal conditions, but also provides useful implications for practices of wetland management and restoration in coastal ecosystems.

## 1 Introduction

Salt marshes are highly productive and biogeochemically active ecosystems that provide critical ecological services, including shoreline stabilization, blue carbon sequestration, nutrient cycling, and habitat provision for coastal biodiversity (Barbier et al., 2011; Duarte et al., 2013; Macreadie et al., 2021; Mcleod et al., 2011; Temmerman et al., 2013). The tidal front zone is a particularly dynamic and vulnerable interface where inundation frequency, wave forcing, substrate instability, salinity stress and nutrient heterogeneity jointly constrain plant survival and growth (Balke et al., 2016; Fagherazzi et al., 2012; Morris et al., 2002). Under such interacting stresses, vegetation often occurs as discrete patches embedded within unvegetated flats, forming irregular, self-organized spatial patterns driven by ‘scale-dependent feedbacks’ (short-range positive feedback and long-range negative feedback) (van de Koppel et al., 2005; Rietkerk et al., 2004; Zhao et al., 2019).

Mostly, these intertidal patches are internally heterogeneous, in terms of both abiotic (micro-habitat) conditions and biotic interactions among plant individuals. Particularly, due to the presence of scale-dependent feedbacks, vegetation patch center is often dominated by stronger positive plant-plant interactions (facilitation) that may affect plant traits therein (Zhao et al., 2019, 2021). Within a patch, the spatial organization of soil properties and plant performance is shaped by local facilitation: established vegetation reduces flow velocity and wave energy (Bouma et al., 2009; van Wesenbeeck et al., 2008; Möller et al., 2014), thus promoting fine-sediment deposition and surface accretion (van de Koppel et al., 2005; Schwarz et al., 2018), further shortening inundation duration and reducing salinity stress, and ultimately enhancing plant growth (van de Koppel et al., 2005). In response to the radially decreasing local facilitation, vegetated patches can exhibit a small-scale gradient (i.e., from patch centers to bare flats) in both soil properties and plant performance. This key role of short-range positive feedback has been supported by transplant experiments showing that aboveground biomass and stem density are consistently higher at patch centers than patch edges (Zhao et al., 2019).

At larger scales, e.g., across the tidal gradient, vegetation patches are embedded in fundamentally different abiotic contexts. Patches at low tidal positions usually experience more frequent inundation, stronger hydrodynamic forcing, and greater sediment exchange, whereas patches at high tidal positions with less physical disturbance often experience greater salinity accumulation, nutrient enrichment, and competitive pressure (Morris et al., 2002; Pennings and Callaway, 1992; Silvestri et al., 2005). These abiotic context differences may alter the primary process through which environmental drivers regulate plant traits. In other words, tidal position may act as a broad environmental filter that shapes not only where patches occur, but also how within-patch soil–trait relationships are represented. Apart from these abiotic processes, how plant-plant interactions vary along tidal gradients has been addressed by the stress gradient hypothesis (Bertness and Callaway, 1994), where facilitative interactions tend to intensify with abiotic stress gradients at broad scales (He et al., 2013). In saltmarsh zones, for example, facilitation among neighboring plants has been found to enhance survival more strongly at low tidal positions that are more physically stressful (Silliman et al., 2015).

Importantly, how these abiotic and biotic factors jointly shape plant traits across broad geographic gradients remains unclear. To address this knowledge gap, plant functional traits offer a mechanistic framework for linking soil properties to plant performance (Lavorel and Garnier, 2002; Violle et al., 2007), serving as suitable proxies for local positive feedback. Typically, leaf dry matter content (LDMC) and specific leaf area (SLA) are closely related to leaf resource-use strategies (Díaz et al., 2016; Wright et al., 2004). In coastal salt marshes, SLA shows a hump-shaped response to inundation, peaking at approximately 30%, whereas leaf dry mass increases monotonically along the same gradient (Wang et al., 2023). Additionally, plant height provides a structural aspect. In tidal marshes, better-aerated and resource-rich sediments generally support greater plant stature and above-ground production (Minden et al., 2012; Schulte Ostermann et al., 2021). In general, these three functional traits respond sensitively to environmental gradients.

Our study focused on salt marshes along the eastern coast of China, where *S. alterniflora* is a widespread invasive species that has displaced native vegetation and formed distinct patchy patterns in tidal front zone. Here, we test three main predictions. First, high and low tidal positions would differ in nutrient availability, sediment texture, and salinity background, and plant height at low tidal positions would be lower and exhibit more conservative leaf strategies (i.e., higher LDMC and lower SLA). Second, at the patch scale, stronger positive feedbacks at patch centers would lead to higher plant heights, lower LDMC, and higher SLA than at patch edges, together with greater nutrient accumulation, finer sediments, and lower salinity. Third, center–edge trait variation would be jointly influenced by variation in soil properties, the surrounding bare-flat matrix, marine environmental conditions, and may serve as a functional indicator of local positive-feedback strength.

## 2 Materials and methods

### 2.1 Study area and sampling design

**Figure 1:**
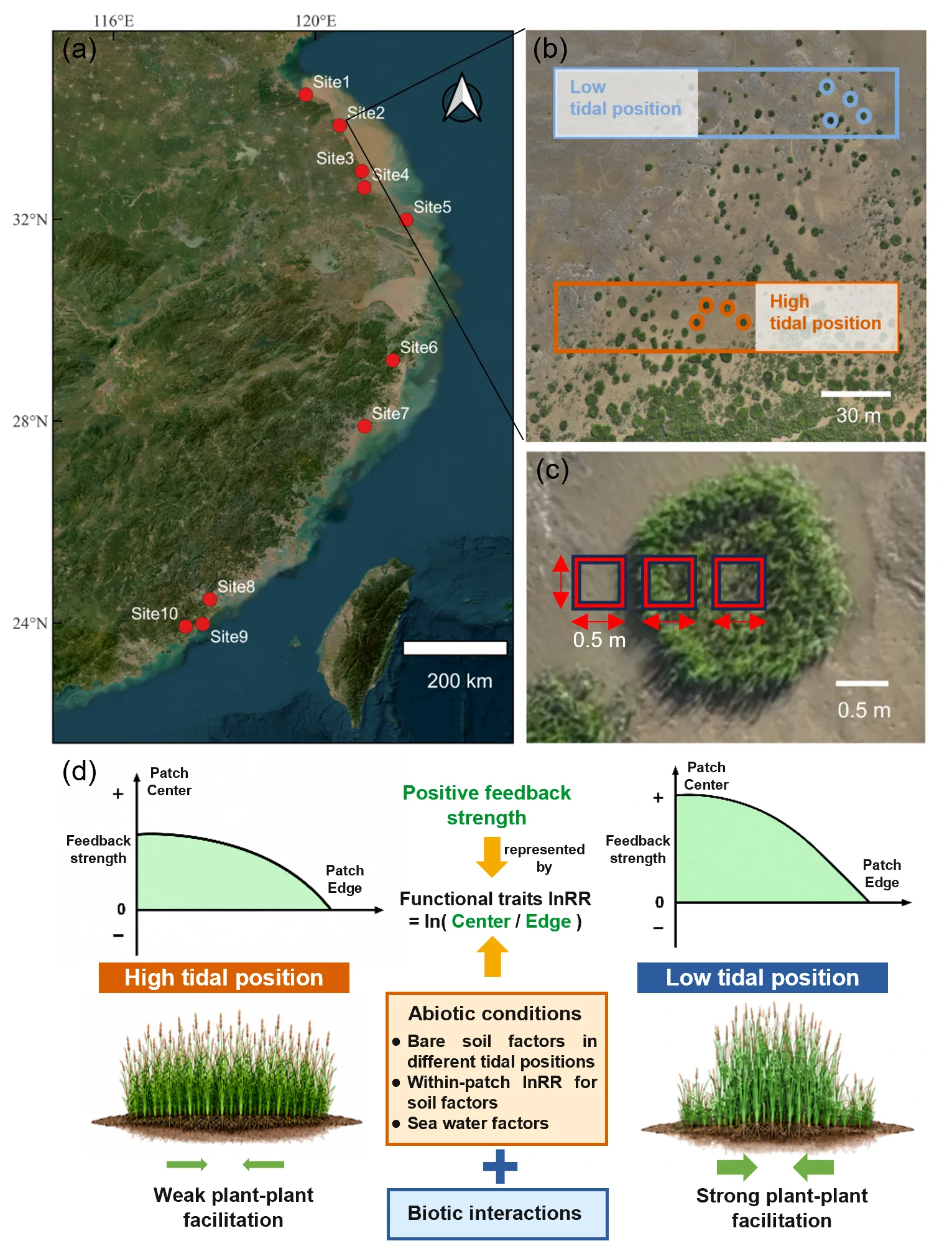
Study sites and sampling design in coastal salt marshes of eastern China. **(a)** Geographical distribution of the 10 study sites across coastal wetlands of eastern China. Red points indicate sampling sites from Jiangsu (1–5), Zhejiang (6–7) and Fujian (8–10) provinces. **(b)** Representative high-resolution UAV-derived orthomosaic from Sheyang, Yancheng, Jiangsu Province (33.86°N, 120.48°E), showing discrete *S. alterniflora* patches under low and high tidal positions. Blue and orange rectangles indicate low and high tidal-position sampling zones, respectively; circles denote four replicated vegetation patches selected within each tidal position. **(c)** Patch-scale sampling design. For each selected vegetation patch, three 0.5 m × 0.5 m quadrats were established along the local center–edge–bare soil gradient. **(d)** Conceptual framework showing the drivers of center–edge functional trait differentiation within vegetation patches.

This study was conducted in coastal salt marshes across the distribution range of *S. alterniflora* in eastern China. 10 sites were selected along a broad latitudinal gradient from 23.93°N to 34.46°N (**Figure 1a**; **Supplementary Table 1**). Field surveys were conducted in Zhejiang Province in October 2023, Jiangsu Province in June 2025, and Fujian Province in September 2025, all during the local active growing period of *S. alterniflora*. To capture within-site tidal heterogeneity, two tidal positions were established at each site: a low tidal position and a high tidal position, separated by at least 50 m (**Figure 1b**). For each tidal position, UAV imagery was acquired and clipped in QGIS 3.30.2 to a 50 m × 50 m window centered on the sampling area. The clipped imagery was imported into eCognition Developer 9.0, where multi-resolution segmentation followed by threshold-based classification was used to distinguish vegetated pixels from non-vegetated ones. Vegetation cover and patch number were then derived for each 50 m × 50 m window (**Supplementary Table 1**).

Within each tidal position, four *S. alterniflora* vegetation patches were randomly selected for field sampling, yielding 80 patches across all sites. For each patch, three 0.5 m × 0.5 m quadrats were established along a patch-scale microhabitat gradient: patch center, patch edge, and adjacent bare soil (**Figure 1c**). The center quadrat was placed in the patch interior, the edge quadrat was located within the vegetated patch boundary, and the bare-soil quadrat was placed on nearby unvegetated sediment within 1 m of the patch margin. Plant measurements were conducted in the center and edge quadrats, whereas soil samples were collected from all three quadrat types. Within each site, all plant and soil samples were collected during the same field campaign following an identical protocol. In total, 160 vegetation quadrats and 240 soil samples were collected across the ten sites.

### 2.2 Measurement of plant traits and soil properties

In each center and edge vegetation quadrat, plant height was measured for 3–10 randomly selected mature and healthy individuals, and the quadrat mean was used in subsequent analyses. For leaf trait measurements, 3–10 mature individuals per quadrat were collected and kept in the dark in black plastic bags at 4 °C for approximately 12 h, with their basal ends immersed in clean water to minimize water loss. After rehydration, fully expanded mature leaves were excised, gently blotted to remove surface water, and weighed to obtain saturated fresh mass. Leaves were then scanned, and leaf area was quantified using ImageJ. The same leaves were oven-dried at 65 °C to constant mass and weighed to obtain dry mass. SLA was calculated as leaf area divided by leaf dry mass (cm²/g), and LDMC was calculated as leaf dry mass divided by saturated fresh mass (Pérez-Harguindeguy et al., 2013).

Soil samples were collected from the 10–30 cm depth layer in patch-center, patch-edge, and adjacent bare-soil quadrats. Our sampling sites were located in intertidal marsh front zones, where frequent inundation and direct exposure to tidal currents make the sediment surface particularly dynamic. Marsh surface sediment deposition can vary substantially among individual tidal events and over short spatial distances, while higher tides may enhance sediment resuspension and delivery to the marsh surface (Ma et al., 2018; Schuerch et al., 2019; Reed et al., 1999). In addition, the physicochemical conditions of surficial intertidal sediments are influenced by repeated inundation, drainage and flushing, as well as evaporation-related changes in salinity (Moffett and Gorelick, 2016; Shen et al., 2018). Considering these disturbance processes present in surficial sediments, we excluded the uppermost 0–10 cm layer and consistently sampled the 10–30 cm depth interval across all sites. Soil samples were collected and analyzed for physical and chemical properties (**Supplementary Table 2**). Samples were air-dried at room temperature, ground, and passed through a 2 mm sieve before laboratory analyses. Bulk density (BD) was determined from undisturbed soil cores using the cutting-ring method. Particle-size distribution was measured by laser diffraction, from which clay, silt, sand fractions and median particle diameter (D50) were derived. Specific surface area (SSA) was calculated from the particle-size distribution assuming spherical particle geometry. Soil electrical conductivity (Ec) was measured potentiometrically in a 1:2.5 soil-to-water suspension using a conductivity electrode. Total nitrogen (TN) was determined by the Kjeldahl method after acid digestion. Total phosphorus (TP) was measured by alkali fusion followed by the molybdenum–antimony colorimetric method, and available phosphorus (AP) was extracted with sodium bicarbonate and determined using the same colorimetric method. Total potassium (TK) was measured by alkali fusion followed by flame photometry, and available potassium (AK) was extracted with ammonium acetate and measured by flame photometry. Nitrate nitrogen (NO_3_^-^–N) was extracted with potassium chloride and determined by ultraviolet spectrophotometry, whereas ammonium nitrogen (NH_4_^+^–N) was extracted with potassium chloride and determined using the indophenol blue colorimetric method.

### 2.3 Data analysis

All analyses were conducted in R (4.5.0). For all linear mixed-effects models (LMMs), patch position, tidal position, and their interaction were fitted as fixed effects, whereas province and sampling site were fitted as random intercepts. Each site was sampled on a single date, and seasonal or phenological offset among sites was absorbed into the site- and province-level intercepts. We examined normality of the predictors and applied log-transformations to LDMC, SLA TP, Ec, NO_3_^-^-N, NH_4_^+^-N and D50 to achieve symmetry and linearity. To characterize bare-soil conditions across tidal positions, we applied PCA to standardized values of TN, TP, TK, AP, NH_4_^+^-N, NO_3_^-^-N, AK, clay, silt, sand, D50, SSA, and BD. PCA was conducted on standardized variables, and PC1 scores were direction-corrected so that higher values represented finer, more resource-rich conditions. Separate models were fitted for ln(LDMC), ln(SLA), and plant height. *Post hoc* comparisons among patch-position × tidal-position groups were based on estimated marginal means with Sidak adjustment. To assess leaf economic coordination, we modelled ln(LDMC) as a function of ln(SLA), group identity, and their interaction, where groups represented the four patch-position × tidal-position combinations. Group-specific slopes were estimated using marginal trends. Radial variation in individual soil variables was analyzed across patch centers, patch edges, and adjacent bare soils.

Center–edge functional trait variation was quantified as log response ratios (lnRR):


$$X\;lnRR=\text{l}n(\frac{X_{Center}}{X_{Edge}})$$

for each trait and soil variable (denoted by *X*), with positive values indicating higher values at patch centers. For each tidal position, trait lnRRs, serving as the external proxy for the positive feedback strength (**Figure 1d**), were regressed against two predictor sets: within-patch soil contrasts (soil lnRRs) and external matrix conditions (adjacent bare-soil properties). Candidate predictors were screened via single-predictor LMMs, then filtered for collinearity (|r| < 0.70, VIF < 5), with no more than three predictors retained per final model. Fixed-effect explanatory power was reported as marginal R² (R^2^m). Marine environmental variables (salinity, dissolved oxygen, nitrate, and phosphate) were obtained from the Bio-ORACLE v2.0 dataset (Assis et al., 2018), representing long-term monthly means (2000–2014) at 5 arcmin resolution.

## 3 Results

### 3.1 Tidal position divergence in bare soil background and patch structure

**Figure 2:**
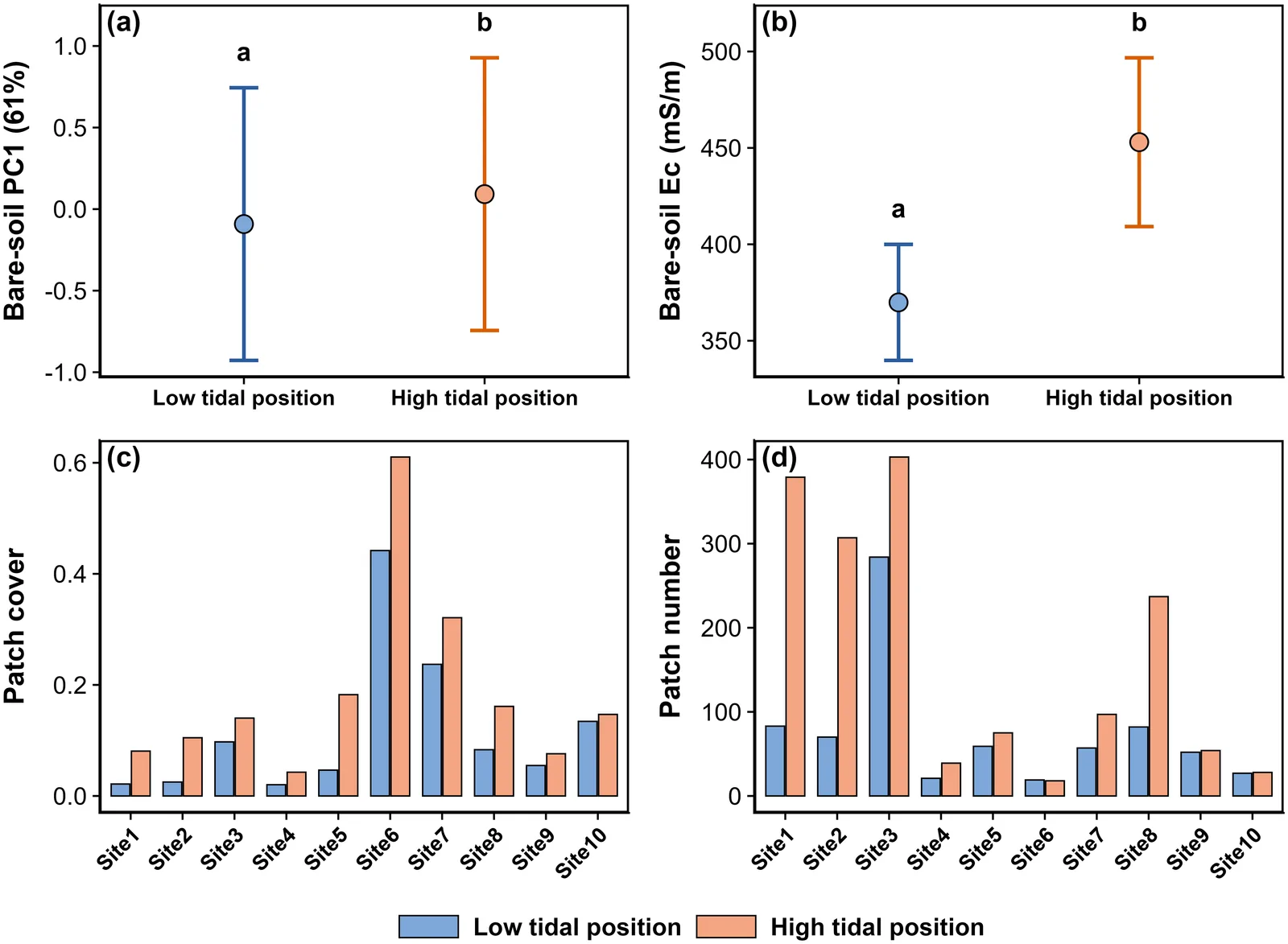
Tidal-position differences in bare-flat soil conditions and patch structure. **(a)** Bare-soil PC1 and **(b)** bare-soil Ec at low and high tidal positions. Points and error bars represent estimated marginal means ± 95% confidence intervals. Different letters indicate significant tidal-position contrasts derived from linear mixed-effects models and subsequent *emmeans* comparisons, rather than significance inferred from overlap between the confidence intervals of individual group means. **(c)** Patch cover and **(d)** patch number across the ten study sites at low and high tidal positions.

Bare-flat environmental conditions diverged clearly between tidal positions (**Figures 2a, b**). High tidal positions had significantly higher bare-soil PC1 and Ec than low tidal positions, indicating a shift towards finer-textured, more saline substrate conditions with higher values along the multivariate soil-resource axis. This PCA-derived axis explained 61% of the total variation and was defined by positive loadings of silt, SSA, clay and several nutrient variables, and negative loadings of sand, D50 and bulk density (**Supplementary Figure 1**). Across the 10 study sites, high tidal positions generally supported greater patch cover and patch number than low tidal positions, although the magnitude of this difference varied among sites (**Figures 2c, d**).

### 3.2 Plant functional traits variation and leaf economic coordination across patch and tidal positions

**Figure 3:**
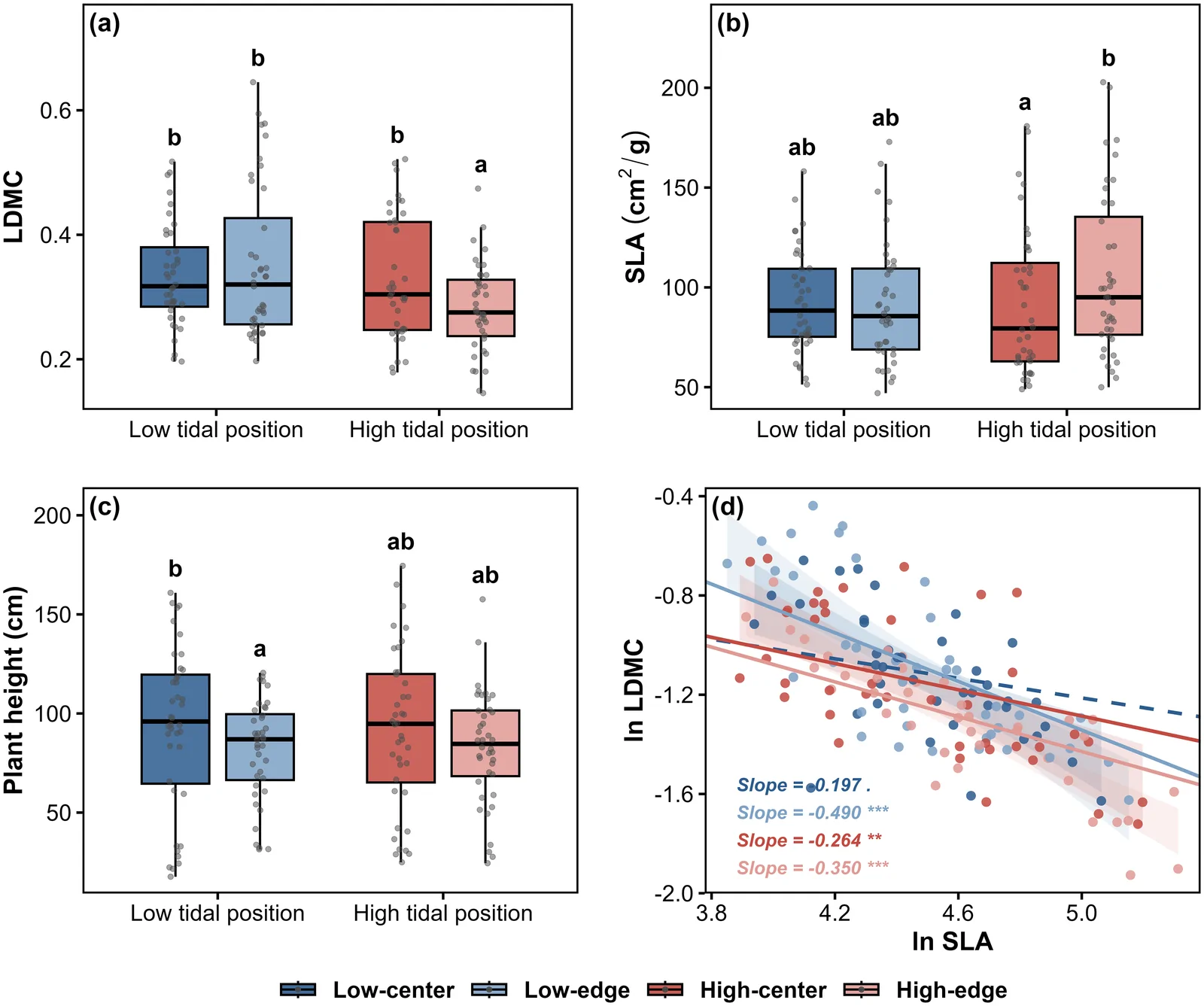
Variation in plant functional traits and leaf economic coordination across patch positions and tidal backgrounds. **(a–c)** Variation in LDMC, SLA, and plant height between patch centers and edges under low and high tidal positions. Regarding Sidak-adjusted *post hoc* comparisons among patch-position × tidal-position groups, no lowercase letter in common indicates significant difference at the 0.05 level, whereas sharing at least one lowercase letter does otherwise. **(d)** Relationship between ln(SLA) and ln(LDMC) across the four patch-position × tidal-position groups. Lines represent fitted group-specific linear relationships, with shaded bands indicating 95% confidence intervals. Solid and dashed lines indicate significant and non-significant slopes, respectively.

The plant functional traits responded differently to patch and tidal positions (**Figure 3**). LDMC showed significant effects of patch position (F = 4.24, *p* = 0.041; **Supplementary Table 3**), tidal position (F = 17.06, *p* < 0.001), and their interaction (F = 10.02, *p* = 0.002), with patch-center plants showing 15.6% higher LDMC than patch-edge plants at the high tidal position. SLA variation was mainly explained by the tidal × patch interaction, with only a marginal patch-position effect and no independent tidal-position effect (F = 2.85, *p* = 0.094). At the high tidal position, patch-center plants had 13.3% lower SLA than patch-edge plants. In contrast, plant height was affected only by patch position (F = 11.73, *p* < 0.001), with no significant tidal position or interaction effect. At the low tidal position, patch-center plants were 13.4% taller than patch-edge plants. The relationship between ln(SLA) and ln(LDMC) was negative across all groups (**Figure 3d**). The negative slope was strongest in low-edge plants (slope = −0.490, *p* < 0.001), followed by high-edge plants (slope = −0.354, *p* < 0.001), high-center plants (slope = −0.264, *p* < 0.01), and low-center plants (slope = −0.197, *p* = 0.078). Thus, the SLA–LDMC relationship remained consistently negative, but its strength varied among patch and tidal contexts, with edge plants showing steeper trait coordination than center plants.

### 3.3 Radial heterogeneity of soil properties from patch centers to bare soil

**Figure 4:**
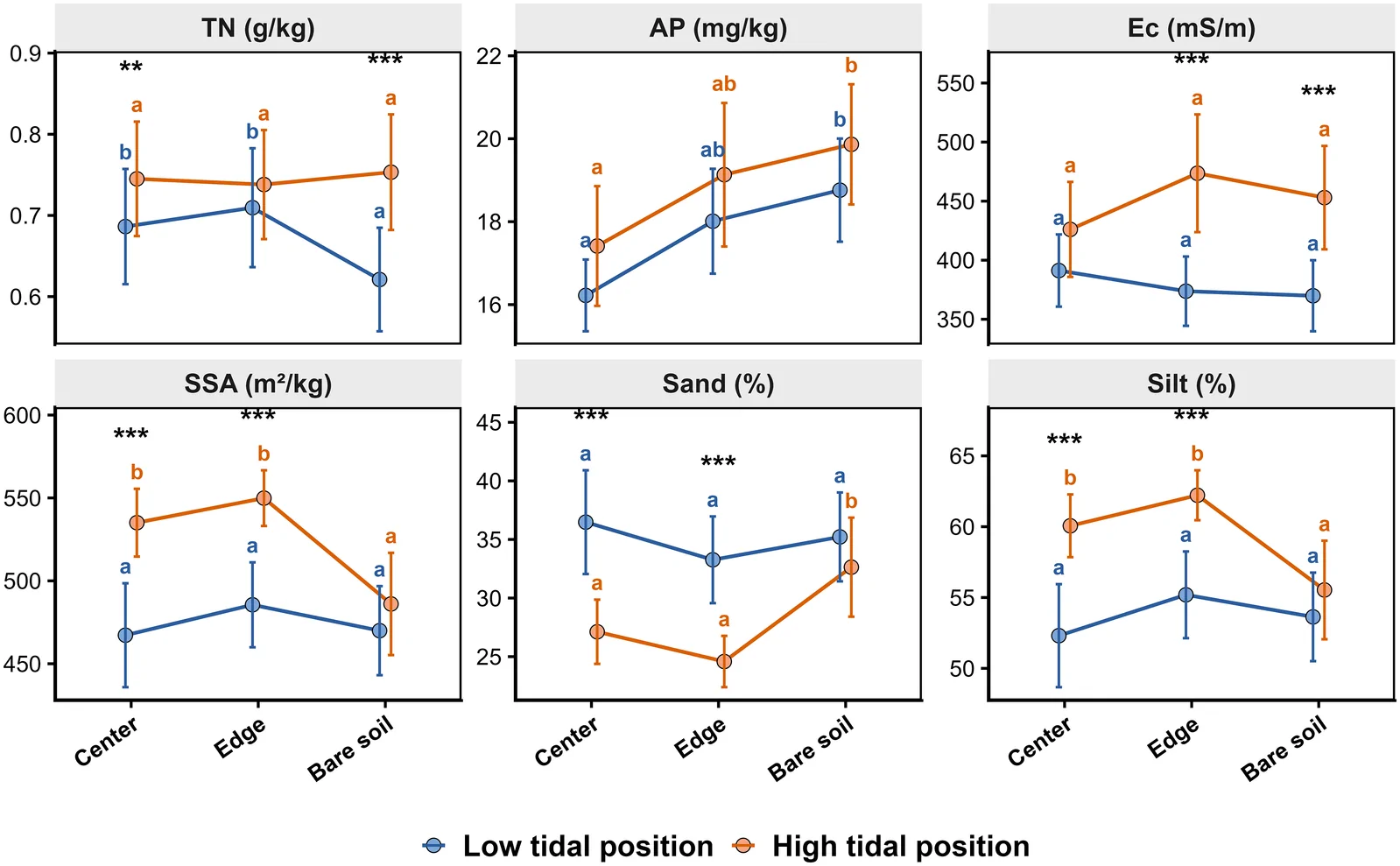
Radial variation in key soil properties across vegetation patches at low and high tidal positions. Points and error bars show group mean ± SE of selected soil nutrient, salinity and sediment properties across patch centers, patch edges and adjacent bare soils at low and high tidal positions. Statistical significance was tested using linear mixed-effects models. Blue and orange symbols represent low and high tidal positions, respectively. No lowercase letter in common indicates significant difference among patch positions for a given tidal position, based on Sidak-adjusted post hoc comparisions. Asterisks indicate significant difference between tidal positions for a given patch position. Significance levels are denoted as ***p < 0.001, **p < 0.01 and *p < 0.05.

The differentiation in soil properties across patch centers, patch edges and adjacent bare soils varied with tidal positions (**Figure 4**). Across all radial positions, high tidal positions generally showed higher TN, AP, Ec, SSA and Silt, but lower Sand content than low tidal positions, indicating consistently finer-textured, more saline and more resource-rich soil conditions at high tidal positions. Within patches, soil properties also varied along the center–edge–bare soil gradient, but the strength of this radial pattern depended on tidal background. At high tidal positions, SSA and Silt were highest at patch edges and declined towards bare soils, while Sand showed the opposite pattern, suggesting stronger sediment fining within vegetated patches. TN remained higher at high than low tidal positions, particularly at patch centers and bare soils, whereas AP increased from patch centers to bare soils in both tidal positions. Ec differed mainly between tidal positions rather than among radial positions, with consistently higher values at high tidal positions.

### 3.4 Within-patch soil–trait links

**Figure 5:**
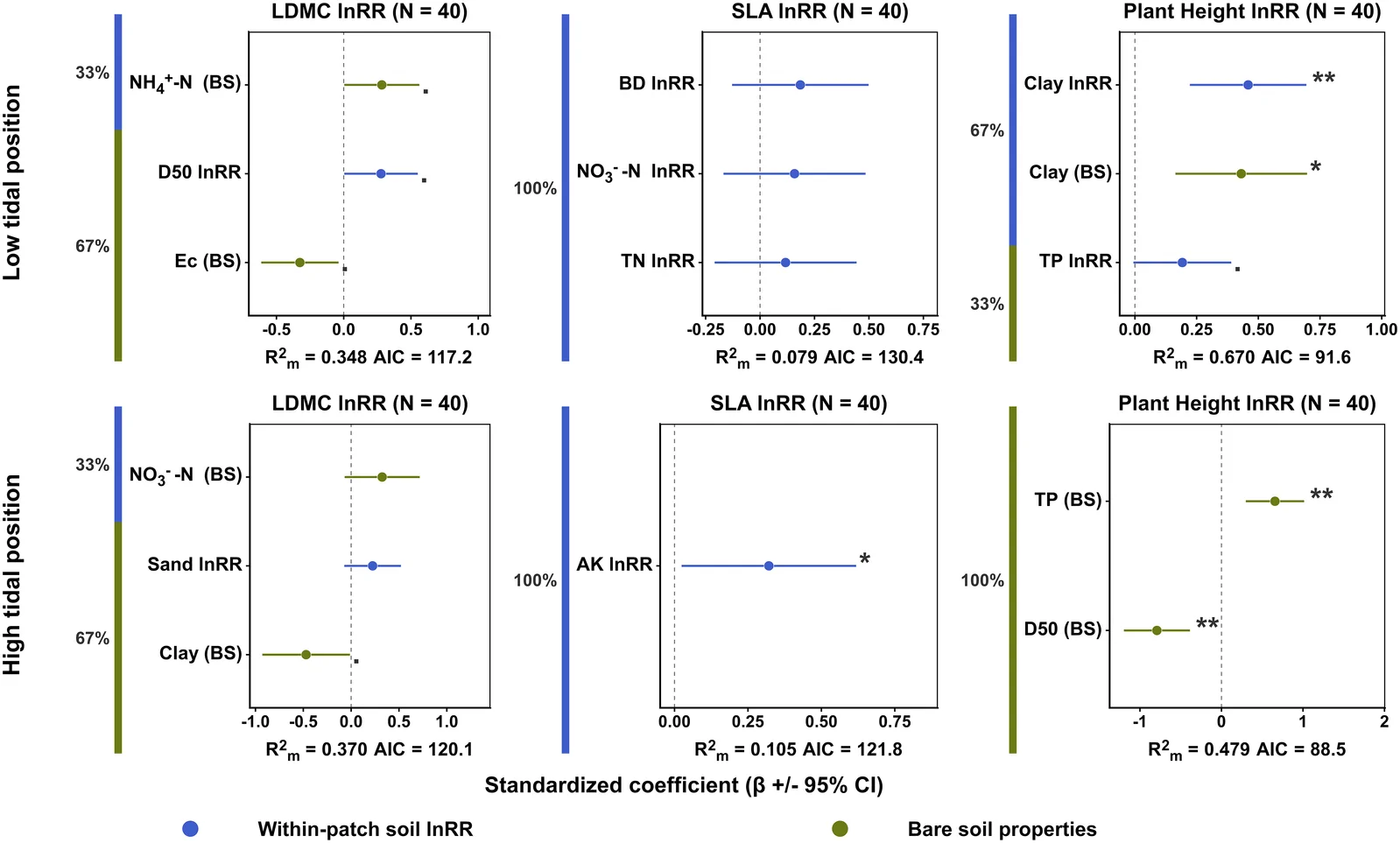
Forest plots showing within-patch soil–trait associations grouped by tidal position. Trait lnRR was calculated as the natural logarithm of the response ratio of patch-center values to patch-edge values. Points indicate standardized regression coefficients, and horizontal lines represent 95% confidence intervals. Blue and green symbols denote patch soil lnRR and bare-soil properties, respectively. Patch soil lnRR refers to the natural logarithm of the response ratio of a given patch center’s soil variables to the patch edge’s, whereas BS indicates bare-soil properties measured outside vegetation patches. Significant effects are indicated by symbols: ***p < 0.001, **p < 0.01, *p < 0.05, and *p* < 0.10. Marginal R² (R^2^m) and AIC are shown for each model.

Within-patch soil–trait links differed markedly between tidal positions and among traits (**Figure 5**). At the low tidal position, plant height lnRR was most strongly explained (R²m = 0.670), increasing with both the within-patch clay gradient (Clay lnRR) and bare-soil clay content, indicating that sediment texture at both internal and external scales could be the main driver of center–edge height variation. Plant height itself also increased with clay content at the low tidal position (R²m = 0.631; **Supplementary Figure 2**), further supporting the role of fine sediments in promoting structural growth. LDMC lnRR was moderately explained by bare-soil nutrients and salinity (R²m = 0.348), with positive effects of bare-soil NH_4_^+^-N and TP. SLA lnRR showed minimal explanatory power (R²m = 0.079). At the high tidal position, explanatory power was generally lower across all traits. Plant height lnRR decreased significantly with bare-soil D50 and increased with TP (R²m = 0.479), while SLA lnRR increased marginally with the within-patch AK gradient (R²m = 0.105). No predictor in the LDMC lnRR model reached significance (R²m = 0.370). Overall, within-patch height differentiation at the low tidal position showed the strongest association with soil conditions. This pattern was shaped not only by internal soil gradients within vegetation patches, but also by the surrounding bare-flat matrix.

### 3.5 Relationships between marine environmental context and center–edge trait variation

**Figure 6:**
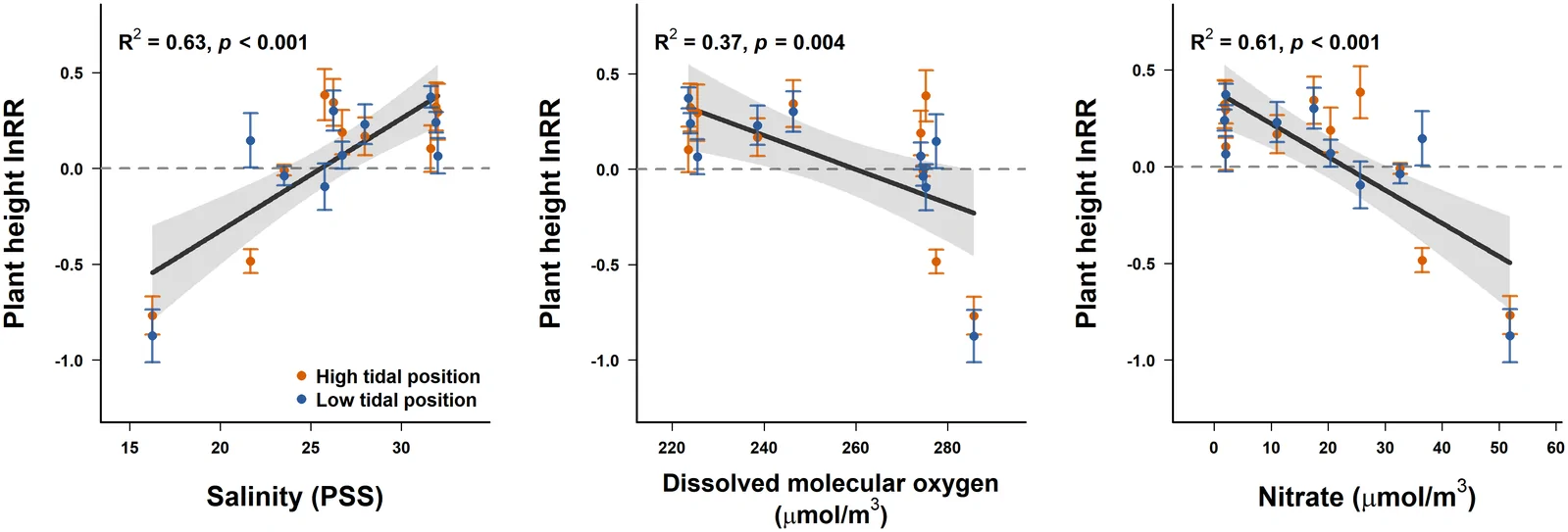
Linear correlations between marine environmental variables and plant height lnRR. Plant height lnRR was calculated as the natural logarithm of the response ratio of patch-center height to patch-edge height. Points show site-level means, and error bars indicate SE. Orange and blue points represent high and low tidal positions, respectively. Black lines show the overall linear regression fitted across both tidal positions, and grey bands indicate 95% confidence intervals. Dashed horizontal lines indicate lnRR = 0, where plant height does not differ between patch center and edge. R² and *p* values are from univariate linear regressions.

Marine environmental variables were strongly associated with plant height lnRR (**Figure 6**), but showed much weaker relationships with leaf economic trait lnRR (**Supplementary Figure 3**). Plant height lnRR increased with salinity (R² = 0.63, *p* < 0.001), but decreased with dissolved molecular oxygen (R² = 0.37, *p* = 0.004) and nitrate (R² = 0.61, *p* < 0.001). These relationships indicate that broader marine environmental gradients were closely linked to the magnitude and direction of center–edge height variation, with more saline and nitrate-poor conditions associated with greater relative height at patch centers. By contrast, LDMC and SLA lnRR showed limited associations with the same marine variables (**Supplementary Figure 3**). LDMC lnRR was weakly related to salinity and nitrate, but showed a modest positive association with phosphate (R² = 0.21, *p* = 0.040). SLA lnRR was not significantly associated with these variables.

## 4 Discussion

In patchy *S. alterniflora* marsh fronts, environmental stress varies multidimensionally across tidal positions. Low tidal positions experience stronger hydrodynamic disturbance and more frequent inundation, which limits nutrient accumulation, whereas high tidal positions show the opposite pattern. Thus, in salt marshes, soil salinity is not necessarily higher closer to the sea. As *S. alterniflora* patches colonize the seaward marsh front, they can enhance sediment deposition and elevate local surfaces, progressively reducing salt input to more landward zones and generating a hump-shaped salinity profile across the saltmarsh (Wang et al., 2022). Consistent with this evidence, our study found higher Ec, a proxy for soil salinity, at high tidal positions than at low tidal positions within patchy front zone.

Tidal position further modulated plant functional traits as a broad environmental filter. Consistent with our prediction, LDMC was higher at low than at high tidal positions, suggesting a more conservative leaf strategy under stronger hydrodynamic disturbance and prolonged inundation. In contrast, SLA did not differ significantly between tidal positions. This lack of a simple tidal contrast is consistent with the hump-shaped response of SLA to inundation (Wang et al., 2023), with maximum values occurring at intermediate flooding frequencies rather than at either end of the gradient. At high tidal positions, where salinity stress was stronger, leaf economic traits showed the greatest sensitivity to local soil conditions (**Supplementary Figure 2**). Elevated salinity can reduce soil water potential and disrupt ion homeostasis, promoting denser and more conservative leaf tissue (Dabravolski and Isayenkov, 2023; Van Zelm et al., 2020). The consistently negative SLA–LDMC relationship across all microsites confirms a leaf economic trade-off (**Figure 3**), but the steeper slope at patch edges, particularly at low tidal positions, suggests that edge environments impose stronger coordination between leaf construction and resource acquisition (Pan et al., 2020; Ries et al., 2004; Violle et al., 2012). Although plants at low-tidal patch edges were shorter, plant height did not show a general decline at low tidal positions despite stronger physical stress (**Figure 3C**, **Supplementary Table 3**), possibly because local facilitation within established patches partially buffered hydrodynamic constraints on vertical growth (Silliman et al., 2015).

At patch scale, contrary to our prediction, patch centers at high tidal positions had higher LDMC and lower SLA than patch edges. One possible explanation is that edge plants occupy more open and dynamic environments, where lower LDMC and higher SLA may facilitate rapid leaf-area deployment and resource acquisition, reflecting a more acquisitive growth or expansion strategy (Pan et al., 2020; Wilson et al., 1999; Wright et al., 2004). Turning to within-patch soil conditions, vegetated patches had finer sediment composition than adjacent bare soils, with higher SSA and silt content but lower sand content at high tidal position (**Figure 4**). Such sediment fining can enhance water and nutrient retention, potentially reinforcing plant-soil feedbacks and contributing to the greater patch cover and number observed at high tidal positions (Temmink et al., 2020; Wilburn et al., 2024). At low tidal positions, by contrast, stronger tidal flushing limited fine-sediment accumulation within patches, patch centers showed evidence of nitrogen accumulation even under stronger tidal exposure. Nitrogen is a primary limiting nutrient in salt-marsh systems, and its enrichment can enhance aboveground production in *Spartina* marshes (Hill et al., 2018; Van Wijnen and Bakker, 1999), consistent with our prediction of greater plant height at patch centers. Across both tidal contexts, available phosphorus was consistently lower in patch centers than in adjacent bare soils, a pattern compatible with rapid uptake or progressive depletion following plant growth (Zhao et al., 2021).

The contrasting within-patch patterns between two tidal positions raise a broader question: what does center–edge functional differentiation represent ecologically? We interpret the trait variation as a functional signature of scale-dependent positive feedbacks, whereby established vegetation ameliorates abiotic stress more strongly at patch centers than at exposed edges. Its magnitude therefore reflects the strength of center-associated environmental buffering (Zhao et al., 2019). In our study, Plant height was the most sensitive indicator of center–edge variation, with clay content emerging as its strongest soil correlate, particularly at low tidal positions (**Supplementary Figure 2**). There, height lnRR increased with both clay lnRR and bare-flat clay content, linking finer sediments to a greater structural advantage at patch centers. Beyond local soil conditions, plant height lnRR increased with ambient marine salinity and decreased with dissolved oxygen and nitrate across sites, indicating that harsher marine environments amplified center-to-edge height variation, which is consistent with the stress gradient hypothesis (Bertness & Callaway, 1994; He et al., 2013), and suggests that plant height lnRR may serve as a scalable functional index for quantifying positive feedback strength across environmental gradients.

By contrast, the measured soil variables did not clearly identify the environmental mechanisms underlying the within-patch differences in SLA. SLA lnRR was weakly explained by the selected soil predictors at both low and high tidal positions (R²m = 0.079 and 0.105, respectively). A broad meta-analysis showed that leaf mass per area, the inverse of SLA, responded most strongly to irradiance, temperature and submergence (Poorter et al., 2009). In salt marshes, SLA has also been shown to be more strongly constrained by salt–waterlogging gradients than by nutrient availability (Minden et al., 2012). This may partly reflect the sensitivity of SLA to light and key hydrological conditions that were not directly quantified in our study. Another possible explanation for the unexpected weak SLA–soil relationship may be related to the soil properties measured only from the 10–30 cm layer, which was meant to correspond to the non-transient nature of plant growth. Indeed, the vertical heterogeneity of marsh soil horizons could be associated with leaf trait development through various soil effects on the root system (Zhao et al., 2024). For the upper 0–15 cm surface sediments, horizontal changes in ammonium and sulfide concentrations have been found to be closely related to plant growth and leaf C:N status (Zhao et al., 2021). For intervals deeper than 30 cm, on the other hand, the relationships between soil physicochemical conditions and leaf traits have rarely been examined. While belowground biomass of *S. alterniflora* has been detected to a depth of 55 cm and some rhizomes may extend beyond 1 m in creek-bank marshes (Reid et al., 2013; Deegan et al., 2012), most live roots are concentrated within the upper 20 cm, which contains approximately 80% of total root biomass (Blum, 1993; Deegan et al., 2012). Overall, it remains unclear about which sediment depth interval is most closely associated with SLA variation.

Overall, our study demonstrates that along the near-tropical to near-temperate geographical gradient, the functional traits of *S. alterniflora* are shaped consistently by the interplay of abiotic constraints and biotic interactions. Moreover, within-patch functional differences, particularly in plant height, can serve as a proxy for local facilitation. This local-scale functional contrast provides a stress-sensitive indicator of how strongly vegetation patches rely on local facilitation to persist under harsh intertidal conditions. These findings offer valuable implications for coastal wetland management and the implementation of Nature-based Solutions (NbS). Although *S. alterniflora* is an invasive species in China, the genus *Spartina* remains one of the most frequently utilized taxa in coastal marsh restoration projects worldwide owing to their strong capacity to establish vegetation and modify sedimentary conditions (Castillo and Figueroa, 2009; Strong and Ayres, 2009; Curado et al., 2020; Rohal et al., 2025; Sloey et al., 2025; Liu et al., 2024). Global meta-analyses indicate that the success of salt-marsh restoration primarily hinges on three key factors: intrinsic species characteristics, abiotic environmental constraints, and biotic interactions (e.g., facilitation, competition and herbivory) (Liu et al., 2024). Among these typical biotic interactions, however, there are hardly feasible approaches to quantifying the strength of facilitation, impeding the link between plant functional traits and wetland-restoration (Sloey et al., 2023). Although our study suggests that within-patch trait differences can suffice to characterize the functional outcome of local facilitation, it is important to bear in mind that the functional dimensions reflecting local facilitation may be taxon-specific. For example, scale-dependent feedbacks in the Chinese native species *Scirpus mariqueter* were associated with changes in shoot-to-root allocation and rhizome branching and extension (Zhao et al., 2019). As climate change and sea-level rise are likely to intensify inundation, salinity stress, and hydrodynamic disturbance, tracking such functional contrasts could help identify marsh areas that are approaching environmental limits and thus require prioritized conservation, restoration, or adaptive management.

## 5 Conclusion

Our study shows that the functional organization of *S. alterniflora* patches is jointly shaped by biotic and abiotic processes operating across tidal, within-patch, and broader marine scales. Tidal position established contrasting bare-flat backgrounds and altered the relationships between plant traits and local soil conditions. Within patches, center–edge trait variation reflected both radial soil differentiation and the influence of the surrounding bare-flat matrix, with leaf economic traits differentiating most strongly at high tidal positions and plant height at low tidal positions. Plant height variation was particularly sensitive to environmental context, indicating that height variation within patches may provide a functional signature of spatial variation in local positive feedback strength. These findings highlight vegetation-patch configuration as an important component of ecosystem engineering and coastal restoration. As sea-level rise and climate change intensify intertidal stress, center–edge height variation may offer a practical signal for identifying marshes nearing environmental thresholds and guiding restoration priorities.

## Data Availability

The raw data supporting the conclusions of this article will be made available by the authors, without undue reservation.
