# Supplementary material for: Environmental stress and plant-plant interactions jointly shape intertidal cordgrass traits across broad spatial scales
Source: Front Plant Sci. 2026 Aug 14;17:1913369. doi: 10.3389/fpls.2026.1913369 (PMC13522210; doi:10.3389/fpls.2026.1913369)
Supplement: Supplementary file 1 [file Table1.docx]

Supplementary Material

# 1 Supplementary Figures and Tables

## 1.1 Supplementary Tables

**Table S1. Supplementary information on the study sites.**

| Region | ID | Longitude  (°E) | Latitude  (°N) | Image Resolution  (m/px) | Patch number  in low tidal position | Cover  in low tidal position | Patch number  in high tidal position | Cover  in high tidal position |
| --- | --- | --- | --- | --- | --- | --- | --- | --- |
| Yancheng | Site1 | 119.81 | 34.47 | 0.028 | 83 | 0.02 | 379 | 0.08 |
| Yancheng | Site2 | 120.48 | 33.85 | 0.029 | 70 | 0.03 | 307 | 0.10 |
| Yancheng | Site3 | 120.92 | 32.95 | 0.031 | 284 | 0.10 | 403 | 0.14 |
| Nantong | Site4 | 120.96 | 32.62 | 0.022 | 21 | 0.02 | 39 | 0.04 |
| Nantong | Site5 | 121.79 | 31.98 | 0.029 | 59 | 0.05 | 75 | 0.18 |
| Ningbo | Site6 | 121.53 | 29.20 | 0.049 | 19 | 0.44 | 18 | 0.61 |
| Wenzhou | Site7 | 120.97 | 27.89 | 0.033 | 57 | 0.24 | 97 | 0.32 |
| Zhangzhou | Site8 | 117.91 | 24.48 | 0.029 | 82 | 0.08 | 237 | 0.16 |
| Zhangzhou | Site9 | 117.77 | 23.98 | 0.029 | 52 | 0.05 | 54 | 0.08 |
| Zhangzhou | Site10 | 117.43 | 23.93 | 0.028 | 27 | 0.13 | 28 | 0.15 |

**Table S2. Detailed descriptions of sample variables.**

| Variable | Description |
| --- | --- |
| Total N (TN) | Sum of all nitrogen forms (organic and inorganic) in soil |
| Total P (TP) | Total phosphorus content in soil, including organic and inorganic forms |
| Total K (TK) | Complete potassium reserves in soil for long-term plant use |
| Ammonium N (NH₄⁺–N) | Ammonium nitrogen in soil that is readily available for plant uptake and microbial assimilation |
| Nitrate N (NO₃⁻–N) | Nitrate nitrogen in soil representing a mobile and plant-available inorganic nitrogen form produced through nitrification |
| Available P (AP) | Phosphorus in soil that can be taken up by vegetation |
| Available K (AK) | Kalium in soil that can be taken up by vegetation |
| Electrical Conductivity (Ec) | Indicators of soluble ion concentrations in soil |
| Bulk Density (BD) | The mass of dry soil per unit volume, reflecting soil compaction and porosity |
| D50 | The particle diameter at the 50th cumulative percentile of the volume distribution. |
| Specific surface area of soil particles (SSA) | The total particle surface area per unit mass |
| Clay | Clay content proportion of fine soil particles smaller than 0.002 mm, influencing water retention, nutrient adsorption, and soil structure |
| Silt | Silt content proportion of soil particles between 0.002 and 0.02 mm, affecting soil texture, fertility, and water-holding capacity |
| Sand | Sand content proportion of coarse soil particles between 0.02 and 2 mm, influencing drainage, aeration, and water retention |

**Table S3. Effects of patch position, tidal position and their interaction on plant functional traits.**

| Response | Effect | numDF | denDF | F | *p* |
| --- | --- | --- | --- | --- | --- |
| LDMC | Patch position | 1 | 150 | 4.24 | 0.041* |
|  | Tidal position | 1 | 150 | 17.06 | < 0.001*** |
|  | Tidal × Patch | 1 | 150 | 10.02 | 0.002** |
| SLA | Patch position | 1 | 150 | 2.85 | 0.094. |
|  | Tidal position | 1 | 150 | 1.13 | 0.289 |
|  | Tidal × Patch | 1 | 150 | 5.59 | 0.019* |
| Plant height | Patch position | 1 | 150 | 11.73 | < 0.001*** |
|  | Tidal position | 1 | 150 | 0.00 | 0.952 |
|  | Tidal × Patch | 1 | 150 | 0.21 | 0.648 |

Note: Models were linear mixed-effects models with province and site as random intercepts. LDMC and SLA were ln-transformed in the models; Plant height was fitted on the original scale. Patch position = Center vs Edge; Tidal position = High vs Low tidal position; Tidal × Patch = their interaction. *p* values are shown with significance codes: *** *p* < 0.001, ** *p* < 0.01, * *p* < 0.05, .  *p* < 0.10.

## 1.2 Supplementary Figures

**Figure S1. Principal component analysis of bare-soil variables.** (a) PCA loading plot showing the distribution of bare-soil variables on PC1 and PC2. (b) Signed loadings of each variable on PC1, ranked by absolute loading magnitude. The PC1 scores were standardized and used in Fig. 2.

**Figure S2. Effects of soil properties and patch position on plant functional traits between low and high tidal positions.** Standardized coefficients (β ± 95% CI) from linear mixed-effects models showing the effects of selected soil variables and patch position on LDMC, SLA, and plant height at low and high tidal positions. Predictor categories are color-coded: green, soil nutrients; blue, soil physical properties; red, Ec; purple, patch position (edge vs. center). Stacked bars indicate the proportional contribution of each category to retained predictors. R²ₘ, marginal R². Significance codes: *** *p* < 0.001, ** *p* < 0.01, * *p* < 0.05, · *p* < 0.10.

**Figure S3.** **Relationships between marine environmental variables and leaf economic trait lnRR.** Panels show the relationships of LDMC lnRR (a–c) and SLA lnRR (d–f) with salinity, phosphate, and nitrate. Points represent site-level means, and error bars indicate SE. Orange and blue points denote high and low tidal positions, respectively. Black lines show overall linear regressions fitted across both tidal positions, with grey bands indicating 95% confidence intervals. Dashed horizontal lines indicate lnRR = 0, where trait values do not differ between patch centers and edges. Reported R² and *p* values are from univariate linear regressions.
